# Hearing colors: an example of brain plasticity

**DOI:** 10.3389/fnsys.2015.00056

**Published:** 2015-04-14

**Authors:** Arantxa Alfaro, Ángela Bernabeu, Carlos Agulló, Jaime Parra, Eduardo Fernández

**Affiliations:** ^1^CIBER-BBNMadrid, Spain; ^2^Hospital Vega BajaOrihuela, Spain; ^3^Department of Magnetic Resonance, INSCANER S.L.Alicante, Spain; ^4^Hospital San RafaelMadrid, Spain; ^5^Institute of Bioengineering, Universidad Miguel HernándezElche, Spain

**Keywords:** functional magnetic resonance imaging (fMRI), diffusion tensor imaging (DTI), magnetic resonance spectroscopy, sensory substitution device (SSD), neuroplasticity, blindness, visual cortex

## Abstract

Sensory substitution devices (SSDs) are providing new ways for improving or replacing sensory abilities that have been lost due to disease or injury, and at the same time offer unprecedented opportunities to address how the nervous system could lead to an augmentation of its capacities. In this work we have evaluated a color-blind subject using a new visual-to-auditory SSD device called “Eyeborg”, that allows colors to be perceived as sounds. We used a combination of neuroimaging techniques including Functional Magnetic Resonance Imaging (fMRI), Diffusion Tensor Imaging (DTI) and proton Magnetic Resonance Spectroscopy (^1^H-MRS) to study potential brain plasticity in this subject. Our results suggest that after 8 years of continuous use of this device there could be significant adaptive and compensatory changes within the brain. In particular, we found changes in functional neural patterns, structural connectivity and cortical topography at the visual and auditive cortex of the Eyeborg user in comparison with a control population. Although at the moment we cannot claim that the continuous use of the Eyeborg is the only reason for these findings, our results may shed further light on potential brain changes associated with the use of other SSDs. This could help to better understand how the brain adapts to several pathologies and uncover adaptive resources such as cross-modal representations. We expect that the precise understanding of these changes will have clear implications for rehabilitative training, device development and for more efficient programs for people with disabilities.

## Introduction

Neuroplasticity is an intrinsic property of the brain, which allows us to adapt to environmental pressures, physiologic changes and experiences by dynamic shifts in the strength of pre-existing connections or by establishing new networks in response to changes in afferent inputs or efferent demands (Pascual-Leone et al., 2005). Consequently, the adult brain has a remarkable capacity to change and adapt throughout the development of lifetime, requiring changes at different levels (genes, molecules, synapses, etc.), and is associated with structural and functional reorganization of the brain (Cramer et al., 2011).

Plasticity can occur during development, learning, in response to the environment or disease, or in relation to therapy. As we interact with a rich multisensory world, our sensory experiences are the product of extensive and dynamic neural connections, which are highly influenced by our experiences and developmental constraints. Such ability can be viewed as adaptivewhen associated with a gain in function (Cohen et al., 1997) or as maladaptive when linked to negative consequences such as loss of function or increased injury (Nudo, 2006). Consequently, the understanding of the nature of these changes is important not only in terms of establishing the brain’s true adaptive potential but also in guiding future rehabilitation strategies (Merabet and Pascual-Leone, 2010).

Sensory substitution devices (SSDs) in people with severe visual impairment offer amazing opportunities to study how the nervous system reorganizes its structure, function and connections when one sensory modality is supplied by another modality. Efficient use of SSDs is based on the brain’s ability to reorganize itself and it depends on the possibility of teaching the brain new complex perceptual skills as well as a different way to process sensory information (Proulx et al., 2014). Although, the most common modality to replace sight is touch, several authors proposed special devices for visual substitution by the auditory pathway in the context of real time reactivity (Meijer, 1992; Capelle et al., 1998; Cronly-Dillon et al., 1999; Striem-Amit et al., 2012; Abboud et al., 2014; Maidenbaum et al., 2014). Sound spatialization consists of virtually creating a three-dimensional auditory environment, where sound sources can be positioned all around the listener. However, these devices often generate unpleasant sensations and lack color information. This motivated the creation of new SSDs that encode color information such as Soundview (Nie et al., 2009), Haptic-Color Glove (Kahol et al., 2006), SeeColOr (Bologna et al., 2009) and EyeMusic (Abboud et al., 2014).

In this framework, our group has had the opportunity to evaluate the brain changes related to the continued use of a visual-to-auditory prosthetic device called *Eyeborg* specifically designed to help color-blind people. This device was developed by Neil Harbisson and Adam Montandon in 2003 and allows colors to be perceived as sound (Eyeborg, 2015). Briefly, it consists of a small video sensor placed at eye level of the head that transmits color information to a computer chip. Electromagnetic light waves are turned into sound frequencies that are heard as musical notes with distinct pitches in real-time (high frequency colors have high notes and low frequency colors have low notes). The computer chip is resting on the back of the head so the sound is heard through vibrations that are directly transmitted into the skull by bone conduction. It is thought that a proficient user of *Eyeborg* could perceive 360 different hues, one for each degree on the color wheel. Each hue is assigned to an audible frequency between 384 and 718 Hz, which allows differentiation between brighter and darker variations as well as color saturation (due to lighting conditions and the environment) and the recognition of the similarities and differences between hues.

In this work, we introduce the potential adaptive and compensatory changes within the brain associated with the continuous use of Eyeborg in a subject with achromatopsia (an irreversible visual impairment characterized by the inability to perceive colors). Our results suggest that after 8 years of continuous use of this device there could be significant changes in functional neural patterns, structural connectivity and cortical topography. Although there is still a lot of work to be done and we cannot claim that the continuous use of the Eyeborg is the only reason for these findings, our study offers the amazing opportunity to delve into the brain’s potential for change by using SSDs. Moreover, it could contribute to a better understanding of the functional and crossmodal organization that supports the neuroenhancement.

## Materials and Methods

### Participants

Five non-synesthete adults (mean 32.2 ± 2.38 years old; males 3, females 2; 4 right handed, 1 left handed) participated in this study. All were enrolled as part of a broader study examining brain plasticity. One of them (the main subject of this study) had difficulty distinguishing colors from childhood and was diagnosed of achromatopsia when he was 11 years old. He is a proficient Eyeborg user that had been using the device, intensively, throughout the whole day during the past 8 years. The control group was an age matched population of healthy volunteers. None of them had absolute pitch. The protocol was approved by the institutional review board and all volunteers gave their written informed consent prior to entering the study.

### Neuroimage Studies

All neuroimaging studies were performed in a Philips Achieva 3T (Philips Medical Systems, Netherlands) with a SENSE Neurovascular coil (18 elements) supplied by the manufacturer. No sedation or contrast agent was given. The protocol included a high-resolution T1-weighted gradient-echo scan: 212 slices, 0.8 mm isotropic voxels, field of view 250 × 250 mm, TR 11 ms and TE 4.9 ms. After the structural data was acquired, functional magnetic resonance (fMRI), diffusion tensor imaging (DTI) and single voxel MR Spectroscopy (^1^H-MRS) studies were carried out.

### Functional Magnetic Resonance Imaging (fMRI)

For the fMRI experiments four different block paradigms were carried out. The visual and auditive stimuli were presented with a special compatible system (VisuaStim Digital Glasses; Resonance Technology, Northridge, CA, USA). Subjects were instructed to keep their gaze fixed on the screen. The tasks were performed in the same order in all the subjects to reduce task interaction effects. No oral output was required avoiding overt movement. fMRI experiment were carried out in the dark.

For the first two paradigms retinotopic mapping stimuli were carried out following conventional procedures based on previously published studies (Engel et al., 1994). The stimuli consisted of high-contrast checkerboards in a rotating wedge and expanding ring. Both stimuli moved in a periodic pattern and completed a full cycle in 24 s with a total of 5 cycles per scanning run. Images were acquired throughout a blood oxygenation level-dependent (BOLD) sensitive T2-weighted multi-slice gradient echo EPI sequence (TE = 35 ms, TR = 2500 ms, FOV = 230 × 230, 64 × 64 matrix). Twentytwo contiguous 4-mm thick axial slices were prescribed parallel to the AC–PC ensuring a full coverage of the occipital lobe. A total of 160 whole brain volumes were collected.

The third paradigm was a color test based on previously published studies (Beauchamp et al., 1999). The stimuli consisted of five wedges arranged around a central fixation bar, extending from one to four degrees of eccentricity. In the chromatic trials subjects were instructed to decide if the colors in the intervening wedges formed an orderly sequence or not. During achromatic trials, wedges of different luminance were presented and subjects decided if the sequence presented a regular luminance sequence or didn’t. The functional scan parameters were as follows: TR = 3000 ms, TE = 35 ms, FOV: 230 × 230, 64 × 64 matrix. Thirty contiguous 4-mm thick axial slices were prescribed parallel to the AC–PC with a total of 84 whole brain volumes collected.

Finally, the forth paradigm consisted of studies where images and sounds were presented simultaneously (Tregellas et al., 2009). The subjects were instructed to look at the images and pay attention to their accompanying sounds, thinking about a putative color that could be assigned to the sound. To encode the sound we used specific algorithms provided by the *Eyeborg* designers. Therefore, we tried to simulate the usage of the Eyeborg inside the environment of the MRI machine. The test presented two condition blocks with a duration of 14 s for each condition. The functional scan parameters were as follows: time repetition = 2000 ms, time echo = 35 ms, FOV: 230 × 230, 64 × 64 matrix. Thirty contiguous 4-mm thick axial slices were prescribed parallel to the AC–PC with a total of 210 whole brain volumes collected.

For the fMRI data analysis, we used SPM8 (The Wellcome Trust Center for Neuroimaging)^1^ implemented in MATLAB 2010a (MathWorks, Inc., Massachusetts). We initially performed motion correction, normalization to the Montreal Neurological Institute (MNI) template, and spatial smoothing (8 mm). After the realignment processes, we checked the head-movement parameters which were not used as covariates in the analyses. The task-related activation was evaluated statistically on a voxel-by-voxel basis using a general linear model at the individual level to generate contrast images, which then were incorporated into analysis at the group level. Statistically significant clusters of activity were recorded for each condition. Only those clusters with a corrected *P* value of 0.05 or less and clusters of 10 voxels or greater are reported. The coordinates reported by SPM (which were in “MNI space”) were transformed to the stereotaxic coordinate frame developed by Talairach and Tournoux (available at http://macdownload.informer.com/talairach-client) and thus enabled comparison to a reference atlas for appropriate neuroanatomical localization. In order to test whether results obtained for our single subject are valid at the population level, we performed a random effects group analysis (RFX) with the help of BrainVoyager QX (Goebel et al., 2006). This neuroimaging software package was also used in same cases for visualization of anatomical and functional data sets.

### Diffusion Tensor Imaging (DTI)

DTI-MR was performed in transversal slice orientation, using a single-shot EPI sequence with diffusion encoding in 32 directions (values 0 and 800 s/mm^2^). The acquired voxel size was 2 × 2 × 2 mm^3^ in 60 slices, with a SENSE factor of 1.9. The diffusion-weighted data were transferred to a workstation for analysis. Before DTI analysis eddy current compensation was applied by affine registration to B0 image. Tractography was then performed using the PRIDE fiber-tracking tool supplied by Philips Medical Systems, as described previously (Nilsson et al., 2007). The ROIs were drawn manually based on the anatomical images and on previously published atlases (Wakana et al., 2004). Tractography was performed based on the connection between two regions of interest (ROI) in order to minimize the risk of including other tracts. Fibers running through the 2 ROIs were computed automatically by the software taking into account a minimum fractional anisotropy value (FA) of 0.3, maximum fiber angle between fibers of 27° and minimum fiber length of 10 mm as stopping criteria.

### Magnetic Resonance Spectroscopy (^1^H-MRS)

For the ^1^H-MRS study the volume of interest was placed based on anatomical structures. The aim was to get an overall spectroscopic representation of different areas within the visual cortex (V1, V2 and V4) avoiding contamination of the surrounding tissues. The resulted volume size was 14 cm^3^ for V1, 12 cm^3^ for V2 and 7 cm^3^ for V4. Shimming and tuning were achieved with automated procedures before acquisition. Water signal was suppressed with selective water signal inversion. For the spectroscopic measurements a standard short-echo-time acquisition mode sequence (TR/TE/averages = 2000/32/128) was used. A total of 512 data points were collected over a spectral width of 1000 Hz. Assignment of the resonances of interest included N-acetylaspartate and other N-acetyl-containing compounds (NAA) at 2.02 ppm, glutamate and glutamine (Glx) at 2.35 ppm, creatine plus phosphocreatine (Cr) at 3.03 ppm, choline and other trimethylamine-containing compounds (Cho) at 3.20 ppm, and myo-inositol (mIno) at 3.55 ppm (Haga et al., 2009). Spectrum analysis was performed off-line with the help of jMRUI 3.0 package (Naressi et al., 2001) available through the MRUI Project^2^ as documented in previous studies (Bernabeu et al., 2009; Poveda et al., 2010).

## Results

All the subjects of the study presented normal MRI images of the brain with neither detectable degenerative changes nor any brain lesions.

### Functional Magnetic Resonance Imaging (fMRI)

Basic visual retinotopic fMRI paradigms showed brain activation in the corresponding visual areas (BA17, BA18 and BA19) in all the subjects studied. No significant differences in the brain activation pattern were observed between the Eyeborg user and the control subjects (data not shown).

**Figure 1:**
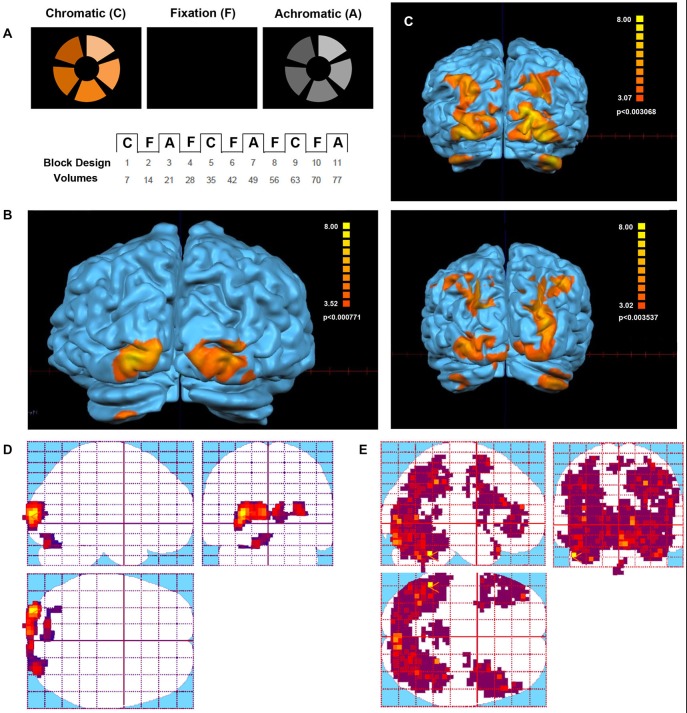
**Visual stimulus and Functional Magnetic Resonance Imaging (fMRI) activation in the Eyeborg user and the control group. (A)** Protocol of the Farnsworth-Munsell test. Within each scan series, the stimulus alternated between blocks of chromatic or achromatic discrimination trials and fixation. **(B)** Areas showing a significant response to chromatic stimulation in the Eyeborg user. **(C)** Areas showing a significant response to chromatic stimulation in two representative controls (single subjects). **(D)** Statistical parametric maps of the Eyeborg user are shown in standard anatomical space. **(E)** Statistical parametric maps of the whole control population in standard anatomical space.

The analysis of the color paradigm showed clear differences between the activated areas of the Eyeborg user and the controls. Particularly, during the chromatic phase of the test, the Eyeborg user showed activation in V1 (BA17) and other small clusters of activation located mainly in the left V2 (BA18). However, the control group showed strong bilateral activation with a great number of other brain areas involved (Figure 1). Thus the color-selective activation in the control group (chromatic blocks vs. fixation) was situated in occipital, temporal, parietal and frontal cortex. More specifically, these areas were located in: (1) occipital lobe (areas BA18 and BA19); (2) temporal lobe related to object and face recognition (BA37), language and auditory processing (BA21, BA22, BA42), memory (BA38) and high level object representation (visual stream BA20); and (3) parietal lobe related to stimulation and texture discrimination tasks (BA40), visuo-motor coordination (BA7, BA6, BA4), and somatosensory functions (BA2); and (4) prefrontal cortex, related with executive functions (BA9, BA10, BA11, 46) and semantic tasks (45, 44). During the achromatic stimulation blocks there was a significant decrease in the number of brain-activated areas in all subjects. In the Eyeborg user we only found activation in areas BA17 and left BA18. In contrast, the control group presented during this achromatic stimulation a higher number of occipital areas activated such as BA17, BA18 and BA19. Moreover, there was brain activation located in the temporal and parietal lobes (BA7 and BA37) and within the entorhinal cortex (BA28) of the right hemisphere.

**Figure 2:**
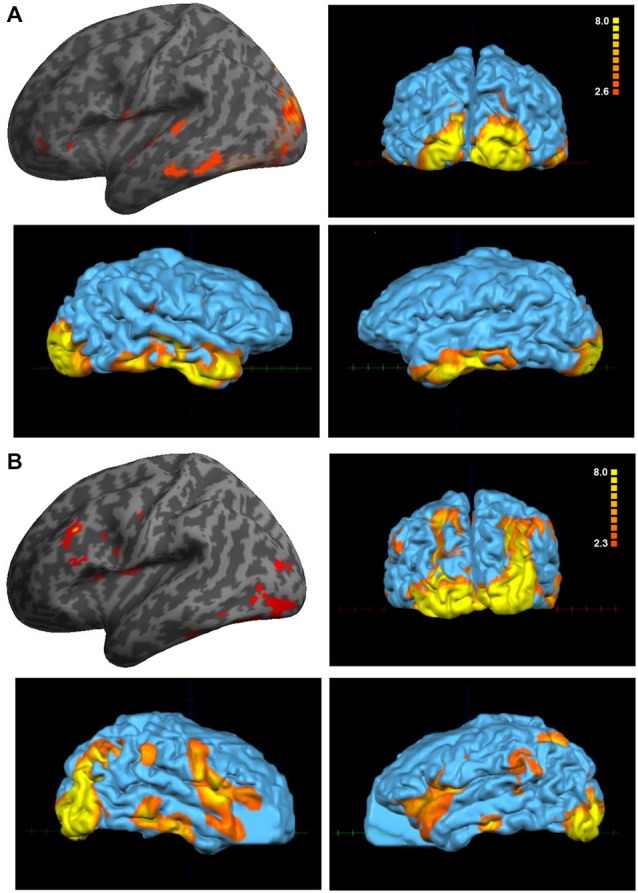
**Analysis of the paradigm involving the presentation of simultaneously visual and auditory stimulation. (A)** Data corresponding to the Eyeborg user (single subject analysis) showing a strong effect on temporal and occipital cortex. **(B)** Averaged activation of the control group in response to the same paradigm of stimulation.

The paradigm that comprised visual and auditory stimuli showed interesting and significant differences between the Eyeborg user and the controls. Particularly, the Eyeborg user displayed a wider brain activation of the auditory pathway and also involved frontal, temporal and occipital areas (Figure 2; Table 1). The frontal activated areas were mainly BA6, BA10 and BA11, which are related to motor and executive functions. The temporal activation was in primary auditory cortex (BA42) and also in areas related to language and auditory processing (BA21) and memory and emotion (BA38). Furthermore, in the Eyeborg user there was a robust activation located in the left BA22 area, which contains Wernicke’s area, and bilateral activation in BA13 (located in the insula) when we presented simultaneously visual and auditory stimuli that were previously encoded by the Eyeborg algorithms.

**Table 1:** **Talairach coordinates and Z-scores of the maximally activated voxels associated with the presentation of simultaneously visual and auditory stimulation**.

		Left	Hemisphere			Right	Hemisphere
	*x*	*y*	*z*	*T* value	Brodman Area	*x*	*y*	*z*	*T* value	Brodman Area
**Eyeborg user**	−9.59	−97.6	18.39	19.4	18	12	−94	22	25	19
	−63.59	−11.2	3.99	15.62	22	33.6	−79.6	−21.2	18.8	18
	−52.79	−0.4	−10.4	11.11	38	69.6	−14.8	0.39	22.58	21
	−34.79	−36.4	18.39	10.09	13	58.8	13.99	−6.8	15.65	38
	−34.79	57.2	−6.8	5.71	10	66	−18.4	18.4	14.69	42
	−27.6	57.2	−3.2	5.53	10	48	−36.4	25.6	8.8	13
						30	50	−14	7.55	11
						22.8	60.79	−6.8	4.74	10
						48	6.79	43.6	6.92	6
						48	3.2	36.4	5.54	6
**Controls**	−38.39	−35.59	−6.8	31.76	47	44.4	−32.8	3.9	181	41
	−45.6	−35.6	14.79	17.99	46	26.4	−79.6	−10.4	169.13	18
	−23.99	17.6	57.9	29.35	6	44.4	3.2	25.6	29.5	9
	−38.4	3.2	54.4	23.45	6	37.2	6.79	25.6	20.74	9
	−16.8	39.2	50.8	19.04	8	44.4	−4	29.2	7.96	6
	−34.79	13	29	24.2	9	40.8	35.6	−14	27	11
	−31.2	−0.4	18.4	16.93	13	44.4	42.79	−3.2	22.3	10
	−42	6	22	20.04	9	66	−40	−14	12.42	21
	−49.2	−54	43.6	9.37	40	48	−29.2	50.8	8.98	40
	−34.8	−54.4	54.4	3.23	7	55.2	−22	36.4	5.47	2
	−49.2	−4	36.4	8.85	6	48	−25	36	5.04	2
	−49.2	−11.2	47.2	6.21	4	33.6	3.2	−17.6	5.62	38
	−42	−7.6	43.6	5.78	6	40.8	28.4	22	7.78	46
	−49.2	28.4	32.8	6.25	9
	−49.2	21.2	36.4	6.08	9
	−52.79	13.99	32.79	3.5	9

*Specification of active clusters at *p* < 0.001 (cluster criterion: 10 voxels)*.

### Diffusion Tensor Imaging (DTI)

We used DTI to trace the microstructure of the main white matter tracts between the brain areas dedicated to auditory and visual processing (temporal and occipital cortices, respectively). By measuring diffusivity in multiple directions, we estimate the FA value. FA is a normalized measure used in diffusion neuroimaging studies which reflects fiber density, axonal diameter, and myelination in white matter (Yoshida et al., 2013). The more aligned fibers are within a tract, the higher the tract or regional FA value, therefore FA differences between groups can be regarded as a surrogate marker of structural adaptation in the white matter (Fabri et al., 2014; Kantarci, 2014).

**Figure 3:**
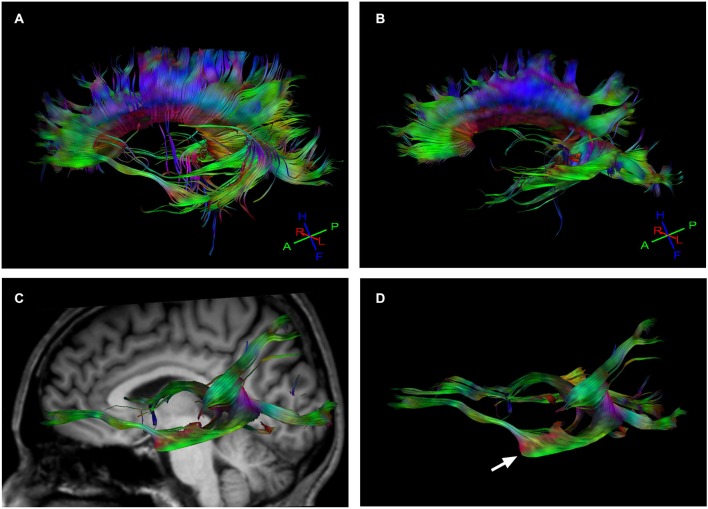
**Tractography results. (A)** Callosal fiber tracks in the Eyeborg user. **(B)** Callosal fiber tracks in a representative control subject (matched in age and sex). **(C)** Left inferior fronto-occipital fasciculus (IFOF), which connects the ventral occipital lobe and the orbitofrontal cortex, in the Eyeborg user. **(D)** Enlargement of the IFOF displayed in panel **(C)**. The arrow points the region with increased myelin density related to the temporal lobe.

When we compared the fiber tracts between the Eyeborg user and the controls we found higher FA values in the Eyeborg user in the corpus callosum (CC), and also (although they were not statistically significant) in both inferior longitudinal fasciculus (ILF) and in both inferior fronto-occipital fasciculus (IFOF) (see Table 2). Neither differences nor interhemispheric asymmetry in the FA or in the apparent diffusion coefficient (ADC) values were observed between groups (data not). In addition to this, by comparing the relative positions and properties of the estimated tracts we found more estimated fiber tracks in the temporal-callosal connections of the Eyeborg user. Furthermore, the IFOF of the Eyeborg user presented a region located in the temporal-basal area with a higher white matter density (Figure 3).

**Table 2:** **Diffusion Tensor Imaging results for white selected white matter tracts in the Eyeborg user and controls**.

		FA value	ADC value (10^−3^MM^2^/S)
***Corpus callosum***	Eyeborg user	0.57 ± 0.02*	0.92 ± 0.03
	Controls	0.52 ± 0.03	0.88 ± 0.02
***Inferior longitudinal fasciculus***
**Left**	Eyeborg user	0.53 ± 0.01	0.85 ± 0.02	
	Controls	0.49 ± 0.03	0.84 ± 0.04
**Right**	Eyeborg user	0.51 ± 0.01	0.86 ± 0.02
	Controls	0.48 ± 0.02	0.82 ± 0.04
***Inferior fronto-occipital fasciculus***
**Left**	Eyeborg user	0.51 ± 0.06	0.82 ± 0.02	
	Controls	0.45 ± 0.08	0.85 ± 0.09
**Right**	Eyeborg user	0.50 ± 0.07	0.83 ± 0.01	
	Controls	0.48 ± 0.01	0.89 ± 0.02

FA = fractional anisotropy value; ADC = apparent diffusion coefficient; *p < 0.05. Diffusion Tensor Imaging results for white selected white matter tracts in the Eyeborg user and controls.

### Magnetic Resonance Spectroscopy (^1^H-MRS)

The mean values and the corresponding standard deviations of the estimated metabolite ratios for the Eyeborg user and the controls are reported in Table 3. There were some differences between the metabolite ratios mIno/Cr, NAA/Cr and Glx/Cr in the visual areas of the Eyeborg user when compared to controls although they were not statistically significant. Specifically, there was an increase in the levels of mIno, a putative marker of glia, in V4 which is the area involved in visual color processing. Furthermore, the Eyeborg user showed an increase in the neuronal integrity marker NAA (NAA/Cr ratio) in V2 and a higher Glx/Cr (Glutamate plus Glutamine) proportion in V1 and V2.

**Table 3:** **^1^H-MRS results showing the metabolite ratios for the Eyeborg user and the control subjects**.

	Metabolite ratios			
Voxel Location	NAA/Cr	Glx/Cr	Cho/Cr	mIno/Cr
**V1**
Eyeborg user	1.53	1.74	0.46	0.42
Controls	1.45 ± 0.12	1.33 ± 0.31	0.52 ± 0.06	0.55 ± 0.09
**V2**
Eyeborg user	1.63	1.27	0.56	0.57
Controls	1.41 ± 0.12	1.05 ± 0.30	0.44 ± 0.01	0.58 ± 0.12
**V4**
Eyeborg user	1.48	0.93	0.43	0.8
Controls	1.44 ± 0.10	1.07 ± 0.17	0.64 ± 0.18	0.62 ± 0.11

*(NAA), N-acetylaspartate and other N-acetyl-containing compounds. (Glx), Glutamate and Glutamine. (Cr), Creatine. (Cho), Choline. (mIno), Myo-inositol. Data obtained at ^1^H MR spectroscopy*.

## Discussion

This study represents the first attempt to directly assess the structural adjustments and neuroplastic changes related to the chronic use of the *Eyeborg*, an electronic device that allows colors to be perceived as sounds. This offers the amazing opportunity to investigate the link between brain activity, conscious perception and technological devices. Our results suggest that after 8 years of continuous use of this device, there could be some adaptive and compensatory changes within the brain. However, we should take into account that this is a very preliminary investigation that needs to be confirmed by further studies with a larger sample size. Thus, some of these changes could exist before the use of the Eyeborg and therefore we cannot claim that the continuous use of the Eyeborg is the only reason of these findings. Nonetheless, several studies have looked into differential activation before and after learning how to use a specific SSD (Ptito et al., 2005; Amedi et al., 2007; Poirier et al., 2007; Connell and Merabet, 2014) showing similar cognitive and neural changes.

The fMRI version of the Farnsworth-Munsell 100 Hue test, requires the use of color information to make perceptual decisions about the sequencing of colors and is able to circumvent the difficulties found in previous studies of color processing areas using passive viewing of colors or simple color discrimination (Beauchamp et al., 1999). Our results showed that the activation in our subject during both, the chromatic and achromatic phases in the color test paradigm, were very similar and constrained to V1 (BA17) and the left V2 area (BA18). This finding fits perfectly with his difficulty for distinguishing between colors and his vision in shades of gray and confirms the usefulness of this test for studying the organization of color-selective areas in normal subjects and in persons with achromatopsia.

The paradigm that comprised visual-auditory stimuli also displayed significant differences in the brain activation pattern of the Eyeborg user. Thus when we presented simultaneously visual images and their correspondent sounds encoded by the Eyeborg algorithms, the activated areas involved the temporal lobe and included the auditory cortex (BA42), Wernicke’s area (BA22) and the rostral part of the superior and middle temporal gyri (BA38), which has been recently related to color and structural judgments of familiar objects (Kellenbach et al., 2005). A particular point of interest is the bilateral activation of BA13 in the Eyeborg user. This finding was not present in any of the control subjects. BA13 is located in the posterior insular cortex and several studies relate this area with phonation, pitch judgment and melodic perception (Zatorre et al., 1994; Kikuta et al., 2006). Moreover, there were robust activations in prefrontal, orbito-frontal, premotor and supplementary motor cortex (BA10, BA11 and BA6). We think that the activation of the orbito-frontal cortex is highly relevant due to its robust connections not only with limbic and olfactory systems but also with the ventral visual pathways involved in the analysis of form and color of the visual information. However, it is also possible that it can be related to some ectopic changes due to other adaptive processes or even to some other preexisting anomalous connections in this particular subject.

The individual variability observed in functional imaging studies may also be related to underlying anatomical patterns that can be studied thanks to recent methodological advances in DTI and fiber tractography (DTI). Our results showed an enhanced structural connectivity between frontal lobe and auditory and visual association areas in the Eyeborg user. Thus, compared to controls, the Eyeborg user has an increased myelin density, which is probably related to a greater number of axons in the IFOF. This is a ventral associative bundle that connects the ventral occipital lobe and the orbitofrontal cortex. Furthermore, IFOF also constitutes one of the major efferent and afferent neuronal projections between the prefrontal cortex, auditory and visual association cortex (Catani et al., 2003; Catani and Mesulam, 2008).

As our subject reports seeing colors upon hearing sounds by using the *Eyeborg*, we hypothesized that the increased FA in IFOF might be related with a pattern of functional connectivity between frontal, auditory and visual association areas and could be involved in enhanced cross-modal associations to process the new information. In support of this idea, an increased structural connectivity in the right IFOF in people with color-music synesthesia has been recently described. This suggests that this perceptual experience could be linked to higher white matter connectivity in this pathway (Zamm et al., 2013). Presently, it remains an open question as to whether, sensory substitution and synesthesia share similar neural mechanisms. In fact, some authors point out that sensory substitution shows properties of synesthesia, as both are associated with atypical perceptual experiences elicited by the processing of a qualitatively different stimulus to that which normally gives rise to that experience (Zamm et al., 2013). In this context, results from the current study seem to suggest that color-sound synesthesia as well as the continued use of the *Eyeborg* could potentially share a common underlying neuroanatomical basis where IFOF has a major role. Another important finding is the higher FA values in ILF in the Eyeborg user. ILF is a direct connection from the occipital cortex to the anterior part of the temporal lobe, running laterally and inferiorly above optic radiation fibers. However, it is important to note that in contrast to IFOF, which is known as a direct pathway (one that connects the occipital, posterior temporal, and the orbito-frontal areas), the ILF is considered to be an indirect pathway that essentially connects similar brain locations. This leads us to believe that IFOF and ILF are probably enhancing similar perceptive processes in our subject, which could be represented by a functional heightened network between frontal, auditory and visual association areas.

The DTI also reveals other structural differences in the Eyeborg user such as an increase of white matter tracts in the CC. The CC is the largest bundle of the human brain and connects left and right cerebral hemispheres. It allows the transfer of inputs from one hemisphere to the other and is involved in several motor, perceptual and cognitive functions (Catani and Thiebaut de Schotten, 2008). In the case of our subject, increased connectivity in CC could be ascribed to the use of *Eyeborg*, however future work will be needed to determine the exact meaning and repeatability of these connectivity changes.

On the other hand, the magnetic resonance spectroscopy (^1^H-MRS) also revealed some changes in metabolic levels of mIno, NAA and Glx in the visual brain areas of the Eyeborg user. Myo-inositol is the most common biological stereoisomer of inositol, a sugar-like molecule synthesized mainly within astrocytes, which has been proposed as a marker of remodeling and plays an essential role in the mechanisms of brain adaptation and plasticity (Bernabeu et al., 2009; Weaver et al., 2013). Specifically, we found an increase in the levels of mIno/Cr in V4, which is mostly involved in color constancy and responds according to perceptual color space. Furthermore, we also found an increase in the NAA/Cr ratio in V2, that is particularly localized within neurons and considered as a major marker for neural integrity. This could reflect not only a higher neuronal and axonal density but also compensatory adjustments in which neurons could be actively implicated. Likewise, there is an increase in the Glx/Cr (Glutamate plus Glutamine) ratio in V1 and V2, in the Eyeborg user which could reflect changes in excitatory biochemical pathways in these areas and a higher synaptic activity (Hensch and Fagiolini, 2005; Bavelier et al., 2010). Taken together all these findings might suggest, the existence of an interconnected network in which glia and neurons work together to process and adapt to the novel sensory experience. In support of this idea, it has been recently proposed (Weaver et al., 2013) that continued use of SSDs might have an effect analogous to the environmental enrichment, resulting in an increase in the astrocyte population and its excitatory synapses that might lead to functional changes in brain activity.

In conclusion, we have tried to present some cues about the possible brain changes associated with the use of the Eyeborg. Our results suggest that there are metabolic, structural and functional changes, particularly in visual and auditive cortices, following the continuous use of this specific SSD device. However, we should emphasize that this is a preliminary work and that further studies, including the analysis of the same subjects before and after the continuous use of a SSD device such as the Eyeborg should be performed. We expect that the precise understanding of these plastic changes will have clear implications for new device development and for more efficient rehabilitative programs for people with disabilities. Furthermore, they could also be helpful in the framework of other approaches for restoring lost neural functions such as innovative artificial vision systems or novel cochlear implants (Fernández et al., 2005; Fernandez and Merabet, 2011).

## Conflict of Interest Statement

The authors declare that the research was conducted in the absence of any commercial or financial relationships that could be construed as a potential conflict of interest.

## Footnotes

^1^http://www.fil.ion.ucl.ac.uk/spm

^2^www.mrui.uab.es/mrui
